# Effect of *NME2* and *SAMHD1* genetic polymorphisms involved in Ara-C metabolism on the response to induction chemotherapy in adult acute myeloid leukemia

**DOI:** 10.1186/s43046-025-00272-4

**Published:** 2025-04-28

**Authors:** Lamiaa Ahmed Fouad, Ghada Mohamed Elsayed, Mosaad M. El-Gammal, Eman Omar Rasekh, Sarah Khaled Ibrahim, Eman Ali Ragab, Fatma B. Rashidi

**Affiliations:** 1 Chemistry Department, Faculty of Science, Biochemistry Division, Cairo University, Cairo, Egypt; 2 Clinical Pathology Department, National Cancer Institute-Cairo University, Cairo, Egypt; 3 Medical Oncology Department, National Cancer Institute-Cairo University, Cairo, Egypt; 4 Chemistry Department, Faculty of Science, Cairo University, Cairo, Egypt

**Keywords:** *NME2*, *SAMHD1*, Polymorphism, Cytarabine, Acute myeloid leukemia

## Abstract

**Background:**

Cytarabine is a prodrug which is activated to cytarabine triphosphate (Ara-CTP) through a series of phosphorylation steps. For considerable leukemic cell death, high level of Ara-CTP is required. Sterile alpha motif and histidine-aspartate domain-containing protein 1* (SAMHD1)* and Nucleotide diphosphate kinase-2* (NME2)* are genes involved in Ara-CTP metabolism. To best of our knowledge, there are no similar studies focused on the association of different polymorphisms involved Ara-C metabolism on the response to induction chemotherapy among adult AML Egyptian patients. Therefore, the aim of this study was to determine the effect of *SAMHD1* rs28372906 and *NME2* rs3744660 polymorphisms on AML complete remission rate (CR), overall survival (OS), and disease-free survival (DFS) among adult AML Egyptian patients, after Ara-C based induction therapy.

**Methods:**

This study was a retrospective conducted at the National Cancer Institute, Cairo University, Egypt. The patient group included 136 adult patients with newly diagnosed AML, while the control group included 48 healthy subjects. The clinical history of all studied patients was collected from patient records. Patients and controls were genotyped for *NEM2* (rs3744660) and *SAMHD1* (rs28372906) by using Taq Man Genotyping assay and Taq Man genotyping master mix (REF: 4,371,353, Applied Biosystems, USA). Real-time PCR assay was performed on Thermo Fisher Quant Studio™ 3. The Statistical Package for Social Science version 21.0 was used to analyze our data.

**Results:**

Regarding the *SAMHD1* (rs28372906) polymorphism, we did not find any genotype variations between patient, and control groups, where all of them were AA genotype. Regarding *NME2* (rs3744660) polymorphism the statistical analysis reported significant association between D28 blasts and OS (*P*-value = 0.043), while the remaining initial patient characteristics and response to induction were not associated with OS.

**Conclusion:**

CR, DFS, and OS were not significantly associated to *SAMHD1* rs28372906 and *NME2* rs3744660 polymorphisms.

## Background

Acute myeloid leukemia (AML) is characterized by the accumulation of aberrant undifferentiated blast cells in the bone marrow [1]. Despite significant advancements in AML medication combination methods and treatment choices, resistance and disease relapse remains a barrier to achieving desired results [2]. Not withstanding the fact that a significant number of patients achieve complete remission (CR) following initial therapy, a small percentage of patients relapse, which has a major impact on long-term survival rates [3].

In healthy young people with AML, the first-line treatment is an aggressive chemotherapy induction followed by one or more consolidation regimens. The traditional intense chemotherapy regimen has been used for decades and consists of three days of chemotherapy followed by three days of rest. Anthracyclines for three days (doxorubicin or idarubicin) and cytarabine for seven days. The rate of complete remission is 60–80%. One or more sessions of high-dose cytarabine are given after intensive therapy to remove residual illness and, if possible, proceed with hematopoietic stem cell transplantation [4]. Subtype-specific therapy is available only for the acute promyelocytic leukemia (APL) M3 [5].

Cytarabine is a prodrug that needs to be converted to Ara-CTP through several steps of phosphorylation processes. For considerable leukemic cell death, high level of Ara-CTP is required. The intracellular concentrations of Ara-CTP are higher in Ara-C sensitive cells than in resistant cells, according to previous in vitro investigations [6].

The sterile alpha motif and histidine-aspartate domain-containing protein 1 (SAMHD1) is a protein responsible for the hydrolysis of Ara-CTP, promoting the detoxification of intracellular Ara-CTP pools [7]. In both cultivated leukemia cells and a mouse model of AML, disrupting *SAMHD1* improves Ara-C sensitivity and cytotoxicity [8, 9].

The nucleotide diphosphate kinase-1 and 2 (*NDPK1/2*, encoded by *NME*1/2) catalyze the last phosphorylation step of Ara-C metabolism leading to active triphosphates of practically all clinically utilized nucleobase and nucleoside analogues [10]. Previous studies have found association between promotor polymorphism in the *NME1/2* and complete response [11].

Genetic polymorphism in the dNTP metabolic pathway involved in Ara-C uptake, activation to Ara-CTP, or inactivation, as well as genes involved in controlling pools of dCTP, have a significant impact on the intracellular level of Ara-CTP and hence its therapeutic efficacy [12, 13].

To the best of our knowledge, there are no similar studies focused on the association of different polymorphisms involved Ara-C metabolism on the response to induction chemotherapy among adult AML Egyptian patients. Therefore,the aim of this study was to determine the effect of different genotypes and alleles of *NME2* (rs3744660) and *SAMHD1* (rs28372906) single nucleotide polymorphism (SNP), on AML CR rate after Ara-C-based induction therapy and investigate SNPs association with overall survival (OS) and disease-free survival (DFS) among adult AML Egyptian patients.

## Methods

This was a retrospective study conducted at the National Cancer Institute (NCI), Cairo University, Egypt. One hundred thirty-six newly diagnosed AML patients from 2016 to 2019 and 48 apparently health control participants, whose ages ranged from 18 to 60 years old, were recruited in the study. Patients who died before day 15 of treatment, those who did not receive Ara-C, poor performance status, significant comorbidities, acute promyelocytic leukemia (M3 subtype), mixed phenotype acute leukemia (MPAL), therapy-related AML, acute lymphoblastic leukemia (ALL), myelodysplastic syndrome (MDS), other hematologic diseases, and solid tumor malignancy were excluded.

### Treatment protocol

All patients were initially treated with the standard “3 + 7” induction therapy approach for adult AML patients using anthracyclines (doxorubicin or idarubicin) and cytarabine: Doxorubicin (DXR) (45 mg/m^2^/day I.V.) or Idarubicin (IDR) (12 mg/m^2^/day I.V.) for 3 days and cytarabine (100 mg/m^2^/day) as a continuous 24 h infusion for 7 days. Three to four courses of cytosine arabinoside (3 g/m^2^ every 12 h on days 1, 3, and 5; total of 18 g/m^2^) were used for consolidation [14].

### Data collection

The clinical history of all studied patients was collected from patients’ records stressing on febrile neutropenia, hepatomegaly, splenomegaly, lymphadenopathy, and central nervous system involvement. Laboratory investigations included initial complete blood count, morphological examination of bone marrow aspirate, immunophenotype, conventional karyotyping, and molecular study for *FLT3*, *NPM1*mutations, t(8;21), and inv(16) translocations as a part of the routine diagnostic work-up of a patient with suspected AML. The 2016 World Health Organization criteria for the categorization of malignancies of hematopoietic and lymphoid organs [13] were used to diagnose and classify. The datasets generated and/or analyzed during the current study are available from the corresponding author on reasonable request.

## Molecular analysis

### Sampling

We collected from 3 to 5 mL of venous blood from 136 AML patients and 48 control participants using sterile ethylenediaminetetraacetic acid (EDTA) vacutainer tube and by venipuncture to genotype them for *NME2* (rs3744660) and *SAMHD1* (rs28372906). The whole blood samples were kept at − 20 °C in the same Vacutainer or used right away for DNA extraction in less than 24 h.

### Extraction of genomic DNA

We extracted the genomic DNA by following the manufacturer’s instruction of Qiagen DNA extraction Mini Kit (GTIN: 04053228005865, LOT: 163,046,931, REF: 51,104, Qiagen, Germany, supplied by Clinilab, Cairo, Egypt). We assayed the DNA quality (concentration and purity) by using Nanodrop2000 spectrophotometer (Thermo-scientific, USA).

### Genotyping

We genotyped the *NME2* (rs3744660) and *SAMHD1* (rs28372906) by using Taq Man Genotyping assay and Taq Man genotyping master mix (REF: 4,371,353, Applied Biosystems, USA). We performed the real-time PCR assay on Thermo Fisher Quant Studio™ 3. We used Thermo Fisher online real-time PCR software for analysis of genotypes (https://www.thermofisher.com/eg/en/home.html).

### Definitions of AML outcomes

Complete remission (CR) was defined by; blasts < 5% in bone marrow, no leukemic blasts in peripheral blood, recovery of peripheral neutrophil counts to more than 1.0 × 10^9^/L, platelet counts to more than 100 × 10^9^/L, and no extramedullary illness [15, 16]. Patient relapse or death is the secondary outcome. Relapse after CR was defined as the presence of at least one of the following: recurrence of more than 5% blasts in the bone marrow and return of leukemic blasts in the peripheral blood [17]. Overall survival (OS) was calculated from the time of admission to the NCI until death from any cause, and it was censored at the last follow-up [15, 18]. Patients who achieved CR had their disease-free survival (DFS) measured from the date of CR to the date of AML relapse or death from any cause, and it was censored at the last follow-up. Until the time of induction, treatment expenditures per patient were reported [15, 19].

### Statistical analysis

The Statistical Package for Social Science (SPSS version 21.0, IBM, USA) was used to analyze the data collected in this study. *P* values less than 0.05 were considered statistically significant. Continuous data was expressed as a median and range, whereas categorical data was expressed as a number and a percentage. Associations of genotypes with CR or non-CR were analyzed by unconditional logistic regression and indicated by odds ratio (OR) and the 95% confidence interval (CI). Survival probabilities were estimated by the Kaplan–Meier method and differences between genotypes was evaluated using the log-rank test. Cox proportional hazard models were constructed for disease-free survival (DFS) and overall survival (OS)**.**

## Results

The study included 136 patients, 69 (50.7%) patients were ≤ 35 years old and 67 (49.3%) were > 35 years. The mean age was 37.7 ± 13.0 years with median of 35.5 years and range from 18 to 60 years. Seventy-five (55.1%) were males and sixty-one (44.9%) were females. The initial clinical patient’s characteristics and laboratory data are illustrated in Tables 1 and 2.

**Table 1:** Baseline clinical characteristics of 136 adult acute myeloid leukemia patients

Parameter	Groups	Number (%)	Parameter	Groups	Number (%)
**Age (years)**	≤ 35	69 (50.7%)	**IPT**	Myeloid	78 (57.4%)
	> 35	67 (49.3%)		Myelomonocytic	58 (42.6%)
**Gender**	Females	61 (44.9%)	**Chemotherapy**	Cytarabine + Idarubicin	57 (41.9%)
	Males	75 (55.1%)		Cytarabine + Doxorubicin	79 (58.1%)
**Organomegaly**	No organomegaly	94 (69.1%)	**FAB groups**	M0	2 (1.5%)
	HM	12 (8.8%)		M1	38 (27.9%)
	HSM	24 (17.6%)		M2	38 (27.9%)
	SM	6 (4.4%)		M4	45 (33.1%)
				M5	13 (9.6%)
**Lymphadenopathy**	Not detected	92 (67.6%)	**Induction**	2 and 5 regimen	3 (2.2%)
	Detected	44 (32.4%)		3 and 7 regimen	133 (97.8%)

*HM* Hepatomegaly, *HSM* Hepatosplenomegaly, *SM* Splenomegaly, *IPT* Immunophenotyping, *FAB* the French-American-British classification of AML

**Table 2:** Laboratory prognostic parameters and remission status in 136 adult acute myeloid leukemia patients

Parameter	Groups	No. (%)	Parameter	Groups	No. (%)
**Hb** **(g/dL)**	≥ 10	16 (11.8%)	**Risk Status according to available karyotype results**	HR	14 (20.6%)
	< 10	120 (88.2%)		IR	50 (73.5%)
**TLC** **(× 10**^**9**^**/L)**	≥ 100	23 (16.9%)		LR	5 (5.9%)
	< 100	113 (83.1%)	***FLT3*-ITD**	Mutant	22 (16.2%)
**PLT** **(× 10**^**9**^**/L)**	≥ 20	35 (25.7%)		Wild	114 (83.8%)
	< 20	101 (74.3%)	***NPM1***	Mutant	25 (27.8%)
**D1 blasts (%)**	≥ 50%	103 (75.7%)		Wild	65 (72.2%)
	< 50	33 (24.3%)	**t(8: 21) detected by RT-PCR**	Negative	111 (88.1%)
**D14 blasts (%)**	≥ 5%	18 (13.2%)		Positive	15 (11.9%)
	< 5	118 (86.8%)	**Inv16(p13;q22)**	Negative	121 (96.0%)
**D28blasts (%)**	≥ 5%	30 (26.7%)		Positive	5 (4.0%)
	< 5	86 (73.3%)	**Overall risk**	HR	29 (21.3%)
**Available karyotype results**	Normal	42 (57.5%)		IR	68 (50%)
	Abnormal	31 (42.5%)			

*Hb* Hemoglobin, *TLC* Total leukocytic count, *PLT* Platelets, *D1* day one of Ara-CTP treatment, *D14* day fourteen of Ara-CTP treatment, *D28* day twenty-eight of Ara-CTP treatment, *RUNX1*: runt-related transcription factor 1, *t(8;21)* translocation (8;21), *RUNX1T1* Runt-related transcription factor 1, translocated to 1, *NA* Not applicable, *HR* High risk, *IR* Intermediate risk, *LR* Low risk, *FLT3*-ITD fms-like tyrosine kinase 3-internal tandem duplication, *NPM1* Nucleophosmin, *RT-PCR* Reverse transcription polymerase chain reaction, *CR* Complete remission

1. Frequency of *SAMHD1* (rs28372906) and *NME2* (rs 3,744,660) genotypes among control and patients’ groups:

As regards the *SAMHD1* (rs28372906), our results showed that all the control and patient samples were of the genotype (AA), and its impact cannot be tested and will not be mentioned anymore. While we revealed that *NME*2 (rs3744660) genotypes among the AML group were 81% GG, 19% were GA and the genotype AA was not detected, while among the control group 81.2% were GG and 18.8% were GA, with no significant association between two groups (*P* = 0.956) (Table 3). Our AML patients were not in Hardy–Weinberg equilibrium.

**Table 3:** Frequency of *NME2* (rs 3,744,660)genotype among control and patients’ groups

Variable	Control group	AML group	*P*-value
GG	39 (81.2%)	110 (81%)	0.956
GA	9 (18.8%)	26 (19%)

2. Association between *NME2* (rs3744660) polymorphism and initial patients’ characteristics:

Logistic regression analysis showed that there was no significant association between *NME2* (rs3744660) polymorphism and different patient characteristics including age (OR = 1.414, 95% CI 0.597–3.352, *P* = 0.431), gender (OR = 1.383, 95% CI 0.577–3.316, *P* = 0.467), and laboratory data (Table 4). Regarding FAB groups, the M1 overall *P*-value was 0.261, where 7 patients (28.0%) were GA and 31 (28.4%) were GG. And regarding high risk (HR), overall *P*-value was 0.320, where 8 (30.8%) were GA and 21 (19.1%) were GG.

**Table 4:** Association between initial patient characteristics and *NME2* (rs3744660) polymorphism

Parameter		**NME2 (rs3744660)**							
		**GA**		**GG**		**95% CI for OR**			
		***N***	**%**	***n***	**%**	**OR**	**Lower**	**Upper**	***P*-value**
**Age groups**	≤ 35 years	15	57.7%	54	49.1%	1.414	0.597	3.352	3.352
	> 35 years	11	42.3%	56	50.9%				
**Sex**	F	10	38.5%	51	46.4%	1.383	0.577	3.316	0.467
	M	16	61.5%	59	53.6%				
**Hb (g/dl)**	≥ 10	3	11.5%	13	11.8%	1.027	0.270	3.905	0.968
	< 10	23	88.5%	97	88.2%				
**TLC × 10**^**9**^**/L**	≥ 100	4	15.4%	19	17.3%	0.871	0.269	2.818	0.817
	< 100	22	84.6%	91	82.7%				
**PLT × 10**^**9**^**/L**	≥ 20	19	73.1%	82	74.5%	1.08	0.41	2.84	0.878
	< 20	7	26.9%	28	25.5%				
**D1 blasts (%)**	≥ 50	19	73.1%	84	76.4%	1.19	0.45	3.15	0.725
	< 50	7	26.9	26	23.6%				
**D14 blast groups**	≥ 5	4	15.4%	14	12.7%	0.802	0.241	2.673	0.720
	< 5	22	84.6%	96	87.3%				
**D28 blast groups**	≥ 5	8	33.3%	23	25.0%	1.50	0.57	3.60	0.411
	< 5	16	66.7%	69	75.0%				
**Lymphadenopathy**	0	21	80.8%	71	64.5%	2.307	0.807	6.597	0.119
	1	5	19.2%	39	35.5%				
**FAB**	M1 vs M2	4	16.0%	34	31.2%	1.409	0.510	3.896	0.509
	M1 vs M4-M5	14	56.0%	44	40.4%	2.705	0.816	8.961	0.104
**IPT**	Myeloid	12	46.2%	66	60.0%	1.750	0.740	4.137	0.202
	Myelomonocytic	14	53.8%	44	40.0%				
***FLT3-*ITD**	Mutant	5	19.2%	17	15.5%	0.768	0.255	2.315	0.639
	Wild	21	80.8%	93	84.5%				
***NPM1***	Mutant	2	10.5%	23	32.4%	4.073	0.867	19.135	0.075
	Wild	17	89.5%	48	67.6%				
**t( 8, 21)**	Negative	22	88.0%	89	88.1%	1.011	0.263	3.895	0.987
	Positive	3	12.0%	12	11.9%				
**Risk**	HR vs IR	13	50.0%	55	50.0%	0.386	0.111	1.338	0.133
	HR vs LR	5	19.2%	34	30.9%	0.622	0.204	1.900	0.405
**CR**	CR	4	30.8%	9	69.2%	2.04	0.58	7.23	0.269
	Non-CR	22	17.9%	101	82.1%				

*F* Female, *M* Male, *Hb* Hemoglobin, *TLC* Total leukocytic count, *PLT* Platelets, *D1* Day one of Ara-CTP treatment, *D14* Day fourteen of Ara-CTP treatment, *FAB* the French-American-British classification of AML, *IPT* thrombocytopaenic purpura, *NA* Not applicable, *FLT3-ITD* Fms-like tyrosine kinase 3-internal tandem duplication, *NPM1 Nucleophosmin*, *HR* High risk, *IR* intermediate risk, *LR* Low risk, *CR* Complete remission

3. Response to induction and survival analysis

All the patients were followed up till day 28 of chemotherapy, 85/136 (62.5%) achieved CR while 31/136 (22.8%) patients were resistant to induction, and 20/136 (14.7%) died before day 28 (Table 2). The cumulative OS and DFS at 2 years for all samples were 55.4% and 38% respectively (Tables 5 and 6).

**Table 5:** Association between different prognostic parameters and overall survival

Parameter	Groups	Number of cases	Number of events	2 years OS (%)	*P*-value
**Genotype**	GA	26	8	60.0	0.267
	GG	110	48	53.9	
**Age groups (ye**a**rs)**	≤ 35	69	25	59.6	0.166
	> 35	67	29	52.3	
**Sex**	F	61	27	47.7	0.178
	M	75	27	61.2	
**Hb groups (gm/dl)**	≥ 10	16	6	61.9	0.649
	< 10	120	48	54.1	
**TLC groups (× 10**^**3**^**/ml)**	≥ 100	23	7	67.1	0.318
	< 100	113	47	53.2	
**PLT groups (× 10**^**3**^**/ml)**	≥ 20	101	37	60	0.103
	< 20	35	17	41.4	
**D1 blast groups D1 blast groups**	≥ 50%	103	41	55.1	0.891
	< 50%	33	13	56.1	
**D14 blast groups**	≥ 5%	40	18	45.4	0.446
	< 5%	95	36	59.2	
**D28 blast groups**	≥ 5%	31	13	45.5	0.043
	< 5%	85	21	71.6	
**Lymphadenopathy**	0	92	37	53.1	0.559
	1	44	17	59.0	
**FAB groups**	M1	38	16	46.4	0.738
	M2	38	15	53.6	
	M4-M5	58	22	61.9	
**IPT diagnosis**	Myeloid	78	32	50.1	0.512
	Myelomonocytic	58	22	61.9	
**FLT3-ITD**	Mutant	22	8	60.2	0.835
	Wild	118	46	54.9	
**NPM1**	Mutant	25	8	66.4	0.677
	Wild	65	23	57.1	
**Trans 8, 21**	Negative	111	39	61.3	0.325
	Positive	15	8	38.9	
**Overall risk**	HR	29	11	61.4	0.933
	IR	68	27	52.3	
	LR	39	18	56.0	

*OS* Overall survival, *F* Female, *M* Male, *Hb* Hemoglobin, *TLC* Total leukocytic count, *PLT* Platelets, *D1* Day one of Ara-CTP treatment, *D14* Day fourteen of Ara-CTP treatment, *FAB* the French-American-British classification of AML, *IPT* thrombocytopaenic purpura, *NA* Not applicable, *FLT3-ITD* Fms-like tyrosine kinase 3-internal tandem duplication, *NPM1 Nucleophosmin*, *HR* High risk, *IR* Intermediate risk, *LR* Low risk

**Table 6:** Association between different prognostic factors and disease-free survival (DFS)

Parameter	Groups	Number of cases	Number of events	2 years DFS (%)	Median	*P*-value
**Genotype**	GA	17	8	38.9	6.4	0.895
	GG	70	34	38.4	16.0	
**Age groups (years)**	< = 35	41	18	48.2	19.5	0.151
	> 35	46	24	26.8	11.6	
**Sex**	F	41	20	36.9	7.7	0.180
	M	46	22	41.6	19.5	
**Hb groups (g/dl)**	≥ 10	9	4	64.8	24.3	0.402
	< 10	78	38	34.8	12.3	
**TLC groups (× 10**^**3**^**/ml)**	≥ 100	15	7	27.4	19.5	0.949
	< 100	72	35	39.3	12.3	
**PLT groups (× 10**^**3**^**/ml)**	≥ 20	65	32	42.6	16.5	0.195
	< 20	22	10	26.1	8.5	
**D1 blast groups**	≥ 50%	63	29	38.4	16.0	0.709
	< 50%	24	13	37.0	8.5	
**D14 blast groups**	≥ 5%	12	4	50.0	12.3	0.191
	< 5%	75	38	37.5	14.9	
**Lymphadenopathy**	0	52	23	41.2	12.3	0.901
	1	35	19	37.8	16.0	
**FAB groups**	M1	19	8	41.2	14.9	0.688
	M2	29	13	38.7	16.5	
	M4-M5	38	23	38.4	11.6	
**IPT diagnosis**	Myeloid	49	19	38.2	14.9	0.486
	Myelomonocytic	38	23	38.4	11.6	
**FLT3-ITD**	Mutant	14	8	22.7	5.1	0.174
	Wild	73	34	42.0	14.9	
**NPM1**	Mutant	20	11	45.7	16.5	0.840
	Wild	37	15	34.4	19.5	
**Trans 8, 21**	Negative	71	32	42.7	16.5	0.727
	Positive	11	6	35.1	11.1	
**Overall risk**	HR	16	9	18.5	14.9	0.119
	IR	42	20	37.2	8.5	
	LR	29	13	48.1	24.3	

*DFS* Disease-free survival, *F* Female, *M* Male, *Hb* Hemoglobin, *TLC* Total leukocytic count, *PLT* Platelets, *D1* Day one of Ara-CTP treatment, *D14* Day fourteen of Ara-CTP treatment, *FAB* the French-American-British classification of AML, *IPT* thrombocytopaenic purpura, *NA* Not applicable, *FLT3-ITD* Fms-like tyrosine kinase 3-internal tandem duplication, *NPM1 Nucleophosmin*, *HR* High risk, *IR* Intermediate risk, *LR* Low risk

4. Association betweenNME2 (rs3744660) genotype and occurrence of AML

Logistic regression analysis showed no significant association between *NME2* (rs3744660) and occurrence of AML (OR = 1.015, 95% CI 0.438–2.354, *P* = 0.972).

5. Association between NME2 (rs3744660) polymorphism and initial patients’ characteristics and response to induction

There was no significant association between *NME2* (rs3744660) polymorphism and different patient characteristics including age (OR = 1.414, 95% CI 0.597–3.352, *P* = 0.431), gender (OR = 1.383, 95% CI 0.577–3.316, *P* = 0.467), complete remission (CR) (OR = 2.040, 95% CI 0.58–7.23, *P* = 0.269) and laboratory data (Table 4).

6. Association between NME2 (rs3744660) polymorphism and OS and DFS

The cumulative OS at two years of 136 patients was 55.4% with 54% death and relapse, while only 87 patients were detected with DFS at 2 years; it was 38% with median of 14.9 and 42% death and relapse. The median OS for age, day 14 blasts, day 28 blasts, and FAB groups were 14.4 months, where it was 7.9 months for platelets groups. The cumulative OS and DFS at 2 years for the GA and GG genotypes was 60.0% and 53.9%, *P* = 0.267, 38.9% and 38.4%, *P* = 0.895), respectively, with no significant differences. There was a significant association between 2 years OS and day 28 blasts < 5 or > 5% (*P* = 0.043), where OS was higher among AML patients with D28 blasts less than 5% than those who were more than or equal to 5% (71.6% and 45.5%, respectively). The remaining initial patient characteristics and laboratory data were not significantly associated with OS. There was no significant association between DFS, regarding initial patient characteristics, and all laboratory data (Tables 5 and 6). The association between D28 and DFS was not calculated, because only three cases were detected to have five or less blasts at day 28 and 84 cases had more than five, these numbers are too small to be compared.

## Discussion

For more than 40 years, cytarabine has been used to treat AML. It is a prodrug that needs to be taken into cells and activated through a series of phosphorylation events to become the drug’s active form [20]. Ara-CTP competes with dCTP for incorporation into DNA, inhibits DNA synthesis, and causes apoptosis [20]. As a result, Ara-CTP intracellular abundance is crucial for therapeutic efficacy, and greater levels may be contributing to observed toxicity and adverse outcomes [20].

Genes involved in Ara-C uptake, activation or inactivation of Ara-CTP, as well as genes involved in controlling pools of dCTP, can affect intracellular Ara-CTP levels and hence therapeutic efficiency. Several studies on the correlation of SNPs within the Ara-C metabolic pathway genes with clinical end points have been published [7–11, 18–25].

For the study of the association between SNP genotyping polymorphism with intracellular Ara-CTP levels in leukemic cells, Elsayed et al. [12] measured the post-initiation of cytarabine infusion in juvenile AML patients, and investigated the genotypes of SNPs in 14 Ara-C metabolic-pathway genes using the Ara-CTP-SNP-score (ACSS).

In this work, we have studied the effect of different genotype and allele frequencies of *NME2* (rs3744660) and *SAMHD1* (rs28372906), SNPs on the response of AML patients to first induction after Ara-C-based therapy; in addition, we have investigated the association with the overall survival (OS) and disease-free survival (DFS).

The ages of our patients ranged from 18 to 60 years, with a mean of 35.22 years. A male predominance was noted (male to female ratio was 1.23:1). The commonest FAB subgroup was M4 (33.1%) followed by M2 (27.9%), and M1 (27.9%). Another study at National Cancer Institute (NCI)-Egypt on 120 adult AML patients showed comparable epidemiological characteristics of Egyptian patients. Their ages ranged between 18 and 86 years with a median of 36.5 years. Male to female ratio was 1.14:1, and the commonest FAB subgroups reported were M4 which represented the most prevalent FAB subtype and accounted for 26.7%, followed by AML-M4 with 25% [26].

CR was achieved in 62.5% of the patients, while 22.8% were not completely remitted, and early treatment mortality was encountered in 14.7% of the studied patients. These results are comparable with Zawam et al. [27], who found that CR was achieved in 57.5% of their studied patients, while 23.8% was not, and early treatment mortality was encountered in 12.5%. However, our study reported higher CR rate, lower non-CR rate, and lower early mortality than that reported in two different Egyptian studies, where El-Zawahry et al. [28] reported that 51% of patients achieved remission, 10% did not, and 39% of patients died very early before evaluation of treatment effect, and recently, Azzazi et al. [29] reported that according to response to chemotherapy in the induction phase, 43.3% were in remission and 36.7% did not respond to chemotherapy and 20% died. The cause behind the different CR rates may be due to different induction regimens.

*SAMHD1* is an Ara-CTPase that can hydrolyze and detoxify intracellular Ara-CTP [30]. Inhibition of the enzyme or CRISPR–Cas9-mediated disruption of *SAMHD1* can sensitize AML cells to Ara-C [31]. In our study, we aimed to analyze the associations of *SAMHD1* SNPs (rs28372906) in the 5′-UTR, with response to induction and AML prognosis. However, all the patients had the reference allele type genotype (AA). We found no significant variation between the control and AML groups. Larger sample size is required to study the effect of *SAMHD1* rs28372906 on the response to Ara-CTP treatment. Zhu et al., 2018 [8] studied the effect of rare *SAMHD1* rs28372906 coding variation on the AML CR rate after Ara-C based induction therapy, the authors reported that there was no significant difference between CR rate and *SAMHD1* rs28372906. Furthermore, Tsesmetzis [11] was able to demonstrate that *SAMHD1* expression levels in adult and pediatric AML patients receiving Ara-C correlate with event-free and overall survival, but not with full remission. The clinical utility of *SAMHD1* genetic variation requires further research to analyze the potential casual variants because the SNP database available contains relatively little SNP information for the *SAMHD1* promoter [8].

The influence of *NME2* (rs3744660) genotype polymorphism on AML was first investigated by Zhu et al. [8]. Association between carriers of this intronic variant *NME2* (rs3744660) polymorphism and response to induction was linked to decreased NME2 mRNA expression, which also indicated decreased DNPK activity and Ara-CTP formation. This finding is consistent with the observation that patients with the rs3744660 A allele have a lower CR rate and that patients with the AA genotype have a worse prognosis. Our results mismatched with that of Zhu et al. [8], who reported that *NME2 (*rs3744660) was observed to be associated with increasing the risk of chemoresistance indicated by CR rate.

We reported that *NME2* (rs3744660) polymorphism was not associated with OS and DFS. These results mismatched with Zhu et al. [8], who reported that among Chinese AML patients, NME2 rs3744660 polymorphism was significantly associated with OS and DFS (*P* = 0.017 and 0.036, respectively), where they were associated with worse OS and DFS. This mismatching may be a result of the difference in ethnicity and larger sample size of Zhu et al. [8] study, where it included 361 AML patients vs. 136 of ours.

For the AML patients involved in our study, genotype distribution for our two SNPs of concern (*SAMHD1* rs28372906 and *NME2* rs3744660) was not in Hardy–Weinberg equilibrium. Similarly, Zhu et al. [8] reported that *SAMHD1* rs28372906 was not in Hardy–Weinberg equilibrium; however, regarding *NME2* rs3744660, they were included in Hardy–Weinberg equilibrium. These may partially be explained by Hardy–Weinberg equilibrium can only be applied for genes that had three genetic models (dominant, recessive, and additive model).

There were some limitations in our study. First is the limited sample size that was as per its availability during the time of the study. Second is that the distribution for *SAMHD1* rs28372906 and *NME2* rs3744660 genotypes in our AML patients were not in Hardy–Weinberg equilibrium, which could be explained in part by the gene’s intrinsic function in the etiology of AML rather than in Ara-c response [8]. Third is depending our discussion on a single study, this was because it is the only similar study to our work. Further studies with large sample sizes among different races are recommended.

## Conclusions

We observed that *NME2* rs3744660 polymorphism was not significantly associated with CR after Ara-C-based therapy, OS, or DFS. However, the *SAMHD1* rs28372906 polymorphism could not be included in the comparisons because all of the studied AML patients and control group ones were wild-type AA.

## Data Availability

The data that support the findings of this study are available from the corresponding author but restrictions apply to the availability of these data which were used under license from the National Cancer Institute for the current study, and so are not publicly available. Data are however available from the corresponding author upon reasonable request.

## Abbreviations

NDPK2, or *NME2*: Nucleotide diphosphate kinase-2
*SAMHD1*: Sterile alpha motif and histidine-aspartate domain-containing protein 1
Ara-CTP: Cytarabine triphosphate
AML: Acute myeloid leukemia
CR: Complete remission
APL: Acute promyelocytic leukemia
dNTP: Deoxynucleoside triphosphate
dCTP: Deoxycytidine triphosphate
SNP: Single nucleotide polymorphism
OS: Overall survival
DFS: Disease-free survival
NCI: National Cancer Institute
MPAL: Mixed phenotype acute leukemia
ALL: Acute lymphoblastic leukemia
MDS: Myelodysplastic syndrome
DXR: Doxorubicin
IDR: Idarubicin
I.V.: Intravenous
*FLT3*: Fms-like tyrosine kinase 3
*NPM1*: Nucleophosmin
DNA: Deoxyribonucleic acid
EDTA: Ethylenediaminetetraacetic acid
GTIN: Global Trade Item Number
REF: Reference
USA: United States of America
PCR: Polymerase chain reaction
SPSS: Statistical Package for Social Science
IBM: International Business Machines
OR: Odds ratio
CI: Confidence interval
ACSS: Ara-CTP-SNP-score
CRISPR: Clustered Regularly Interspaced Short Palindromic Repeats
mRNA: Messenger ribonucleic acid
